# Molecular epidemiology of the protective RV144 V2 loop epitope

**DOI:** 10.1186/1742-4690-9-S2-P320

**Published:** 2012-09-13

**Authors:** T Cardozo

**Affiliations:** 1 NYU School of Medicine, New York, NY, USA

## Background

The immune correlate analysis of the RV144 trial identified antibodies (Abs) targeted at the V1-V2 domain as potentially protective vaccine responses. Reactivity of vaccinee serum Abs with a glycosylated gp70-V1-V2 fusion protein or with certain V2 loop peptides was significantly associated with a lower risk of viral infection. A V2 loop segment approximately comprising amino acid residues 165-178 of gp120 was also identified by pilot vaccinee studies as a key immunogenic region.

## Methods

To identify whether the segment identified by pilot studies coincides with the reagents reactive in the case control study, I mapped the locations of the reagents associated with lower risk of viral infection onto the V1-V2 domain structure. With a single segment identified as associated both with immunogenicity in the pilot studies and protection in the case control study, I calculated the Dayhoff evolutionary sequence distance between the the sequences of this segment in the immunogens and in a panel of V2 loop based reagents used in the case control study, each with associated odds ratios (OR) for risk. OR was then plotted against distance.

## Results

The same V2 loop segment from positions 165-178 that was specifically most immunogenic in the pilot studies appears to have been associated with protection in the immune correlate analysis. This segment corresponds to the "C" strand of the V1-V2 domain beta-sheet fold, exactly where the broadly neutralizing antibody PG9 binds. Plotting the evolutionary distance between this segment in the RV144 immunogens and synthetic V2 loop based antigens used in the case control study shows that lower risk (lower OR) correlates with greater evolutionary distance.

## Conclusion

A V2 loop segment from positions 165-178 of gp120 was highly immunogenic in humans. Abs elicited by the RV144 subtype E immunogen displaying segment may have been protective only if they cross-reacted distantly with subtype B.

